# The prognostic value of visual and automatic coronary calcium scoring from low-dose computed tomography-[^15^O]-water positron emission tomography

**DOI:** 10.1093/ehjci/jeae081

**Published:** 2024-03-26

**Authors:** M M Dobrolinska, R A Jukema, S G M van Velzen, P A van Diemen, M J W Greuter, N H J Prakken, N R van der Werf, P G Raijmakers, R H J A Slart, P Knaapen, I Isgum, I Danad

**Affiliations:** Department of Radiology, Medical Imaging Center, University of Groningen, University Medical Center Groningen, Groningen, The Netherlands; Department of Nuclear Medicine and Molecular Imaging, Medical Imaging Center, University of Groningen, University Medical Center Groningen, Groningen, The Netherlands; Department of Cardiology, Amsterdam UMC, Vrije Universiteit Amsterdam, Amsterdam, The Netherlands; Department of Biomedical Engineering and Physics, Amsterdam UMC location University of Amsterdam, Amsterdam, The Netherlands; Informatics Institute, University of Amsterdam, Amsterdam, The Netherlands; Amsterdam Cardiovascular Sciences, Amsterdam, The Netherlands; Department of Cardiology, Amsterdam UMC, Vrije Universiteit Amsterdam, Amsterdam, The Netherlands; Department of Radiology, Medical Imaging Center, University of Groningen, University Medical Center Groningen, Groningen, The Netherlands; Department of Nuclear Medicine and Molecular Imaging, Medical Imaging Center, University of Groningen, University Medical Center Groningen, Groningen, The Netherlands; Department of Robotics and Mechatronics, Faculty of Electrical Engineering, Mathematics & Computer Science, University of Twente, Enschede, The Netherlands; Department of Radiology, Medical Imaging Center, University of Groningen, University Medical Center Groningen, Groningen, The Netherlands; Department of Nuclear Medicine and Molecular Imaging, Medical Imaging Center, University of Groningen, University Medical Center Groningen, Groningen, The Netherlands; Department of Radiology, University Medical Center Utrecht, Utrecht, The Netherlands; Department of Radiology and Nuclear Medicine, Amsterdam UMC, Vrije Universiteit Amsterdam, Amsterdam, The Netherlands; Department of Radiology, Medical Imaging Center, University of Groningen, University Medical Center Groningen, Groningen, The Netherlands; Department of Nuclear Medicine and Molecular Imaging, Medical Imaging Center, University of Groningen, University Medical Center Groningen, Groningen, The Netherlands; Department of Biomedical Photonic Imaging, Faculty of Science and Technology, University of Twente, Enschede, The Netherlands; Department of Cardiology, Amsterdam UMC, Vrije Universiteit Amsterdam, Amsterdam, The Netherlands; Department of Biomedical Engineering and Physics, Amsterdam UMC location University of Amsterdam, Amsterdam, The Netherlands; Informatics Institute, University of Amsterdam, Amsterdam, The Netherlands; Amsterdam Cardiovascular Sciences, Amsterdam, The Netherlands; Department of Radiology and Nuclear Medicine, Amsterdam UMC location University of Amsterdam, Amsterdam, The Netherlands; Department of Cardiology, Amsterdam UMC, Vrije Universiteit Amsterdam, Amsterdam, The Netherlands; Department of Cardiology, University Medical Center Utrecht, Heidelberglaan 100, Utrecht 3584 CX, The Netherlands

**Keywords:** coronary calcium score, computed tomography, low-dose CT scans, positron emission tomography, coronary artery disease

## Abstract

**Aims:**

The study aimed, firstly, to validate automatically and visually scored coronary artery calcium (CAC) on low-dose computed tomography (CT) (LDCT) scans with a dedicated calcium scoring CT (CSCT) scan and, secondly, to assess the added value of CAC scored from LDCT scans acquired during [^15^O]-water-positron emission tomography (PET) myocardial perfusion imaging (MPI) on prediction of major adverse cardiac events (MACE).

**Methods and results:**

Five hundred seventy-two consecutive patients with suspected coronary artery disease, who underwent [^15^O]-water-PET MPI with LDCT and a dedicated CSCT scan were included. In the reference CSCT scans, manual CAC scoring was performed, while LDCT scans were scored visually and automatically using deep learning approach. Subsequently, based on CAC score results from CSCT and LDCT scans, each patient’s scan was assigned to one out of five cardiovascular risk groups (0, 1–100, 101–400, 401–1000, >1000), and the agreement in risk group classification between CSCT and LDCT scans was investigated. MACE was defined as a composite of all-cause death, non-fatal myocardial infarction, coronary revascularization, and unstable angina. The agreement in risk group classification between reference CSCT manual scoring and visual/automatic LDCT scoring from LDCT was 0.66 [95% confidence interval (CI): 0.62–0.70] and 0.58 (95% CI: 0.53–0.62), respectively. Based on visual and automatic CAC scoring from LDCT scans, patients with CAC > 100 and CAC > 400, respectively, were at increased risk of MACE, independently of ischaemic information from the [^15^O]-water-PET scan.

**Conclusion:**

There is a moderate agreement in risk classification between visual and automatic CAC scoring from LDCT and reference CSCT scans. Visual and automatic CAC scoring from LDCT scans improve identification of patients at higher risk of MACE.

## Graphical Abstract

**Graphical Abstract:**
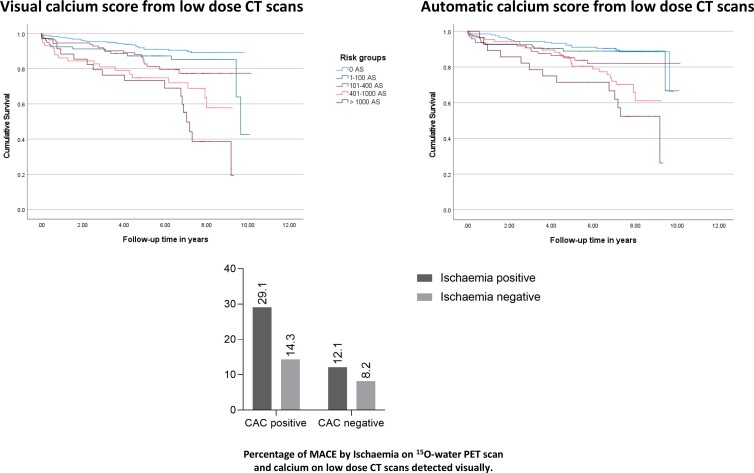

## Introduction

Coronary artery calcium (CAC) is a marker of atherosclerosis and a well-known cardiovascular risk predictor for both asymptomatic individuals and symptomatic patients^1,2^ and was implemented in clinical guidelines as a risk modifier.^3,4^ Importantly, in clinical practice, CAC scoring follows the strictly defined Agatston method which is applicable to electrocardiogram (ECG)-triggered, non-contrast calcium scoring computed tomography (CSCT) scans.^5^ In daily clinical practice, low-dose CT scans (LDCT) instead of CSCT are performed in adjunction to myocardial perfusion positron emission tomography (PET) for attenuation correction. The importance of CAC as a risk modifier highlighted the potential for LDCT scans to ascertain the presence of calcium.^3,4^ Based on the Society of Cardiovascular Computed Tomography and the Society of Thoracic Radiology (SCCT/STR) guidelines, CAC from LDCT scans should be reported.^6^ Several LDCT CAC scoring methods are described, including a visual analysis with high accuracy when using CSCT as the gold standard.^7^ Recent artificial intelligence applications led to proliferation of studies that describe automatic LDCT scan calcium scoring methods, which offer alternatives for the time-consuming manual scoring.^8–10^ Currently, no agreement has been reached on which method is preferred.^6^

In clinical practice, patients with a high likelihood of myocardial ischaemia are evaluated with functional non-invasive testing.^3^ Within myocardial perfusion imaging (MPI) methods, [^15^O]-water-PET is considered the gold standard for myocardial blood flow (MBF) quantification.^11^ Nevertheless, MPI ^15^O-water-PET exclusively provides information about perfusion without offering information on the presence of non-obstructive atherosclerotic plaques within the coronary arteries.

It has been shown that information regarding the presence of coronary calcium adds clinical value to MPI scans.^12^ The combination of MPI and CAC from LDCT improves detection of significant stenosis.^13^ Moreover, the amount of CAC from dedicated CSCT scans also helped to determine patients at higher cardiovascular risk.^14–16^ However, the value of CAC measured from LDCT scans of MPI [^15^O]-water-PET has not been studied, yet.

Therefore, the aim of our study is two-fold: firstly, to validate automatically and visually scored CAC on LDCT using a dedicated CSCT as reference and, secondly, to assess the prognostic value of CAC score from LDCT scans acquired during [^15^O]-water-PET MPI for the prediction of long-term major adverse cardiac events (MACE).

## Methods

### Study design and image acquisition

**Figure 1:**
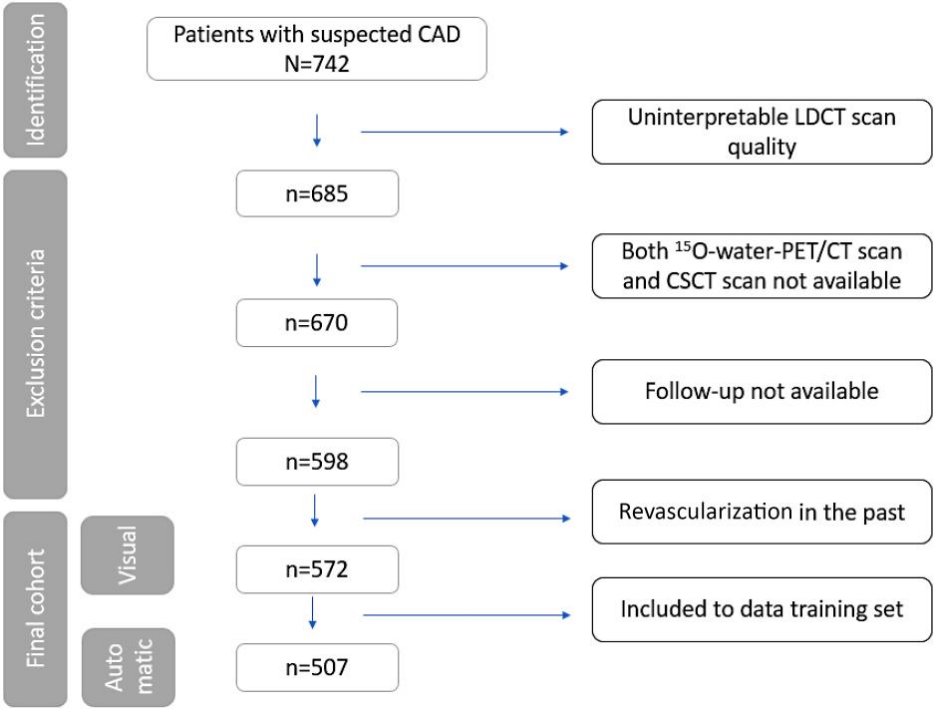
Flowchart of included patients.

In this single-centre study, 742 consecutive patients with suspected CAD, who underwent [^15^O]-water-PET MPI with LDCT and a dedicated CSCT scan, were included between 2008 and 2014 (*Figure 1*). Both scans were acquired on the same day. Detailed information on patient’s inclusion criteria and image acquisition parameters is given in Supplementary data online, *Material*. The need for written informed consent was waived by the institutional review board (Medical Ethics Committee of the Amsterdam UMC, Vrije Universiteit Amsterdam) due to the retrospective study design.

### CAC scoring from LDCT scans

#### Visual

For visual scoring of both rest and stress LDCT scans, a previously described five-point scale patient risk group was used: 0, 0; 1, 1–100; 2, 101–400; 3, 401–1000; and 4, >1000 Agatston score (Supplementary data online, *Figure S1*).^7^

#### Automatic

**Figure 2:**
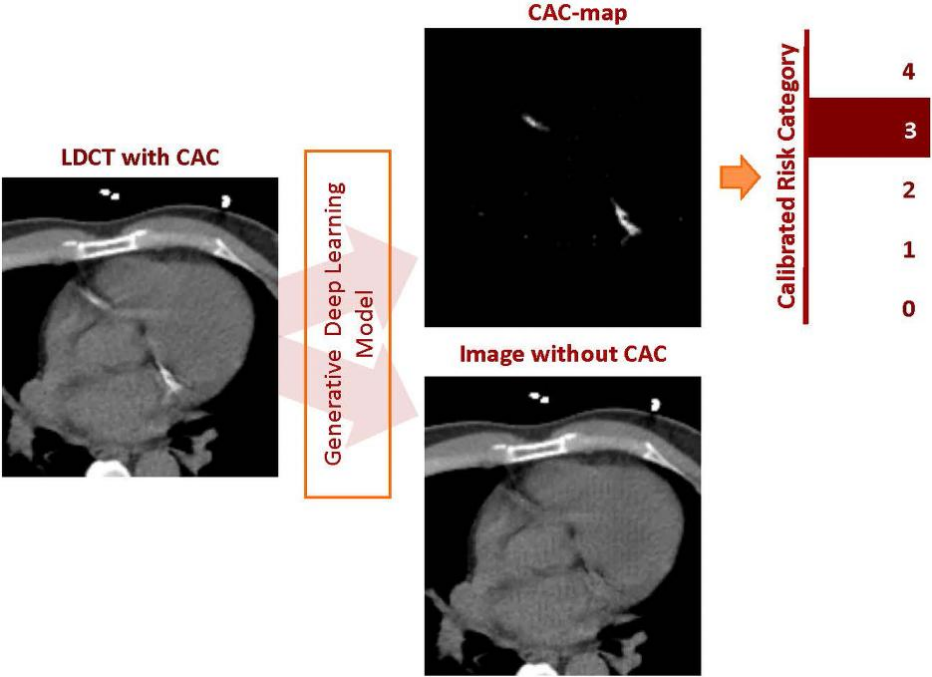
Schematic overview of the image decomposition into the image without CAC and a CAC map.

For automatic scoring, a previously described method was applied^17^ and is summarized in Supplementary data online, *Material*. Based on automatic scores, scans were defined as calcium negative and calcium positive. As a next step, calcium-positive scans were assigned a cardiovascular risk category based on calculated CAC pseudomass.^17^ Cardiovascular risk categorization was based on a five-point scale that was calibrated using visual scores and defined as follows: 0, 0; 1, 1–100; 2, 101–400; 3, 401–1000; and 4, >000 Agatston score (*Figure 2*).^17^ The groups were equivalent to the five-point scale used in visual scoring.

### Study endpoint

Follow-up data were obtained using a national registry database, medical records, and telephonic contacts. The follow-up data were tracked from the date of PET scan until 1 February 2019. For the current analysis, the study endpoint was defined as all-cause mortality, non-fatal myocardial infarction (MI), coronary revascularization (percutaneous coronary intervention or coronary artery bypass graft surgery), and unstable angina. Revascularizations were adjudicated, and early revascularizations resulting from the initial non-invasive imaging were excluded from this composite outcome.

### Statistical analysis

For categorical variables, frequencies, percentages, and proportions were calculated. For continuous variables, either means (with standard deviation) or medians (with interquartile ranges, IQR) were calculated. Normality was assessed visually based on histograms and Q–Q plots. Clinical characteristics between CAC-positive and CAC-negative groups based on LDCT were compared using Student’s *t*-test or Wilcoxon rank-sum test (for continuous values) and χ^2^ (for categorical values).

The agreement in calcium risk group classification for visual and automatic calcium scoring on LDCT with the reference (manual calcium scores on CSCT) was quantified using Cohen’s linearly weighted κ with 95% confidence intervals (95% CI). The sensitivity, specificity, positive predictive value (PPV), and negative predictive value (NPV) of visual and automatic LDCT calcium scoring (CAC > 0 vs. CAC = 0) were calculated.

Kaplan–Meier curves with log-rank tests were plotted to analyse the event-free survival of patients stratified for CAC detected on LDCT and CSCT. The association between coronary calcium detected on LDCT and time-to-first MACE was assessed with univariable Cox proportional hazard model and presented as hazard ratio (HR) with 95% CI. The multivariable Cox proportional hazard model included PET results (ischaemia vs. no ischaemia) and the CAC scored from LDCT or CSCT. Visually and automatically assessed CAC on LDCT and manually scored CAC on CSCT were tested in separate multivariable Cox proportional hazard models. Harrell’s *C*-index was used to compare the predictive performance of three multivariable Cox regression models which included results of PET perfusion scan and one of the three CAC scoring methods, respectively (model 1, [^15^O]-water-PET and CSCT scan; model 2, [^15^O]-water-PET and visual scoring from LDCT scans; and model 3, [^15^O]-water-PET and automatic scoring from LDCT scans). A *P* value of <0.05 was considered statistically significant. All statistical analyses were performed using SPSS 28 and SAS 9.4 M8.

## Results

### Patient characteristics

Five hundred seventy-two patients were included with a mean age of 58 ± 9 years (*Table 1*). During 6.6 ± 1.9 years of follow-up, 16.7% of patients experienced MACE (late revascularization, 6.9%; MI, 3.5%; unstable angina, 1.4%; death, 4.9%). Of all included patients, the median CSCT Agatston score was 50 (IQR: 0.0, 307.0). Based on PET and visual LDCT calcium scoring, 182 (31.8%) patients had ischaemia on PET and CAC, 91 (15.9%) had ischaemia but no CAC, 105 (18.4%) were free from ischaemia but CAC-positive, and 194 (33.9%) presented with no ischaemia and no CAC. Patients with CAC on LDCT were older (62 ± 8 vs. 55 ± 9 years, *P* < 0.001), more frequently male (67.4% vs. 40.4%, *P* < 0.001), and had more comorbidities (hypertension, *P* < 0.001; hypercholesterolaemia, *P* = 0.005; diabetes mellitus type II, *P* = 0.003), as compared with patients with a CAC-negative LDCT. The global hyperaemic MBF was also significantly lower in patients with a CAC-positive LDCT (3.4 ± 1.2 vs. 2.5 ± 1.1 mL/min/g, *P* < 0.001).

**Table 1:** Patients’ characteristics by visual CAC scoring from LDCT scan

	Overall	LDCT CAC-negative	LDCT CAC-positive	*P*
	(*n* = 572)	(*n* = 285)	(*n* = 287)	
**Baseline**				
Age (yrs)	58 ± 9	55 ± 9	62 ± 8	<0.001
Male	309 (54.0)	115 (40.4)	194 (67.4)	<0.001
BMI	27.1 ± 4.1	27.1 ± 4.5	27.0 ± 3.8	0.064
Statin therapy	381 (66.6)	160 (56.1)	221 (77.0)	<0.001
ASA	424 (74.1)	187 (65.5)	237 (82.6)	<0.001
Beta-blocker	354 (61.9)	164 (57.5)	190 (66.2)	0.056
ARB	103 (18.0)	40 (14.0)	63 (22.0)	0.016
ACEI	115 (20.1)	50 (17.5)	65 (22.6)	0.174
Diabetes mellitus type II	109 (19.1)	40 (14.0)	69 (24.0)	0.003
Hypertension	269 (47.0)	111 (38.9)	158 (55.1)	<0.001
Current smoker	193 (33.7)	91 (31.9)	102 (35.5)	0.424
Hypercholesterolaemia	212 (37.1)	89 (31.2)	123 (42.9)	0.005
Family history of CAD	298 (52.1)	152 (53.3)	146 (50.9)	0.446
Chest pain				0.257
Typical	169 (29.5)	76 (26.7)	93 (32.4)	
Atypical	200 (35.0)	98 (34.4)	102 (35.5)	
Non-specific	194 (33.9)	104 (36.5)	90 (31.4)	
**Follow-up**				
Dyspnoea	48 (8.4)	19 (6.7)	29 (10.1)	0.175
Elective CABG and PCI	47 (8.2)	15 (5.3)	32 (11.1)	0.014
Revascularization based on initial work-up	106 (18.5)	17 (6.0)	89 (31.0)	<0.001
Revascularization not based on initial diagnostic work-up	60 (10.5)	16 (5.6)	44 (15.3)	<0.001
Early PCI	65 (11.4)	14 (4.9)	51 (17.8)	<0.001
Early CABG	41 (7.2)	3 (1.1)	38 (13.2)	<0.001
MACE	95 (16.6)	27 (9.5)	68 (23.7)	<0.001
PCI late	51 (8.9)	15 (5.3)	36 (12.5)	0.003
CABG late	12 (2.1)	1 (0.4)	11 (3.8)	0.006
MI	20 (3.5)	5 (1.8)	15 (5.2)	0.038
Unstable angina	8 (1.4)	1 (0.4)	7 (2.4)	0.068
Mortality	28 (4.9)	8 (2.8)	20 (7.0)	0.031
**Imaging data**				
Calcium score—gold standard	50.0 (0.0, 307.0)	0.0 (0.0, 12.8)	299.4 (114.7, 624.5)	<0.001
PET perfusion imaging				
Global sMBF	2.95 (1.22)	3.4 ± 1.2	2.5 ± 1.1	<0.001
LAD sMBF	1.92 (1.26)	3.3 ± 1.2	2.5 ± 1.1	<0.001
RCA sMBF	2.90 (1.29)	3.3. ± 1.3	2.5 ± 1.1	<0.001
LCx sMBF	3.05 (1.23)	3.4 ± 1.2	2.6 ± 1.1	<0.001
Ischaemia	273 (47.7)	91 (31.9)	182 (63.4)	<0.001

ACEI, angiotensin-converting enzyme inhibitor; ARB, angiotensin receptor blocker; ASA, acetylsalicylic acid; BMI, body mass index; CABG, coronary artery bypass graft; CAD, coronary artery disease; LAD, left anterior descending artery; LCx, left circumflex artery; LDCT, low-dose CT scan; MACE, major adverse cardiac event; sMBF, stress myocardial blood flow; MI, myocardial infarction; PCI, percutaneous coronary intervention; PET, positron emission tomography; RCA, right coronary artery.

### CAC analysis from LDCT

#### Visual analysis

The risk classification agreement between reference manual CSCT and visual LDCT was 0.66 (95% CI: 0.62–0.70) (*Table 2*). A total of 57.2% of the LDCT scans were assigned to the same risk category as reference scans. Of patients incorrectly assigned as CAC-negative, 89.6% (112/125) had an Agatston score of 1–100 based on the reference CSCT scan (*Table 2*). Sensitivity, specificity, PPV, and NPV of CAC detection on LDCT scans was 68.8%, 95.8%, 97.6%, and 55.8%, respectively (*Table 4*).

**Table 2:** Agreement in risk classification between CSCT scan and visual CAC scoring from LDCT scans

		Visual scoring from LDCT scans					
		0	1	2	3	4	Total
CAC scoring from CSCT scan	0	160	6	1	0	0	167
	1	112	44	8	2	1	167
	2	11	27	69	16	1	124
	3	1	3	30	22	2	58
	4	1	0	5	18	32	56
	Total	285	80	113	58	36	572

Weighted Kappa 0.66 (95% CI: 0.62–0.70). Risk groups were defined as follows: 0, 0 AS; 1, 1–100 AS; 2, 101–400 AS; 3, 401–1000 AS; and 4, >1000 AS.^7^

**Table 3:** Agreement in risk classification between CSCT scan and automatic CAC scoring from LDCT scans

		Automatic scoring from LDCT scan					
		0	1	2	3	4	Total
CAC scoring from CSCT scan	0	124	20	5	3	2	154
	1	69	37	26	10	3	145
	2	5	35	43	25	1	109
	3	0	2	20	23	3	48
	4	0	0	4	27	20	51
	Total	198	94	98	88	29	507

Weighted Kappa 0.58 (95% CI: 0.53–0.62). Risk groups were defined as follows: 0, 0 AS; 1, 1–100 AS; 2, 101–400 AS; 3, 401–1000 AS; and 4, >1000 AS.^7^

**Table 4:** Sensitivity, specificity, PPV, and NPV of CAC detectability on LDCT scans based on visual and automatic scoring, as compared with a reference, CSCT scan

	Visual LDCT	Automatic LDCT
Sensitivity (%)	68.8	79.3
Specificity (%)	95.8	80.4
Positive predictive value	97.6	90.3
Negative predictive value	55.8	62.7

#### Automatic analysis

The agreement in risk classification between reference CSCT manual scoring and automatic scoring from LDCT was 0.58 (95% CI: 0.53–0.62) (*Table 3*). Of patients incorrectly assigned as CAC-negative, 93.2% (69/74) had an Agatston score of 1–100. The sensitivity and specificity of CAC detection based on automatic LDCT CAC scoring was 79.3% and 80.2%, respectively (*Table 4*).

### Prognostic value of CAC on MACE

**Figure 3:**
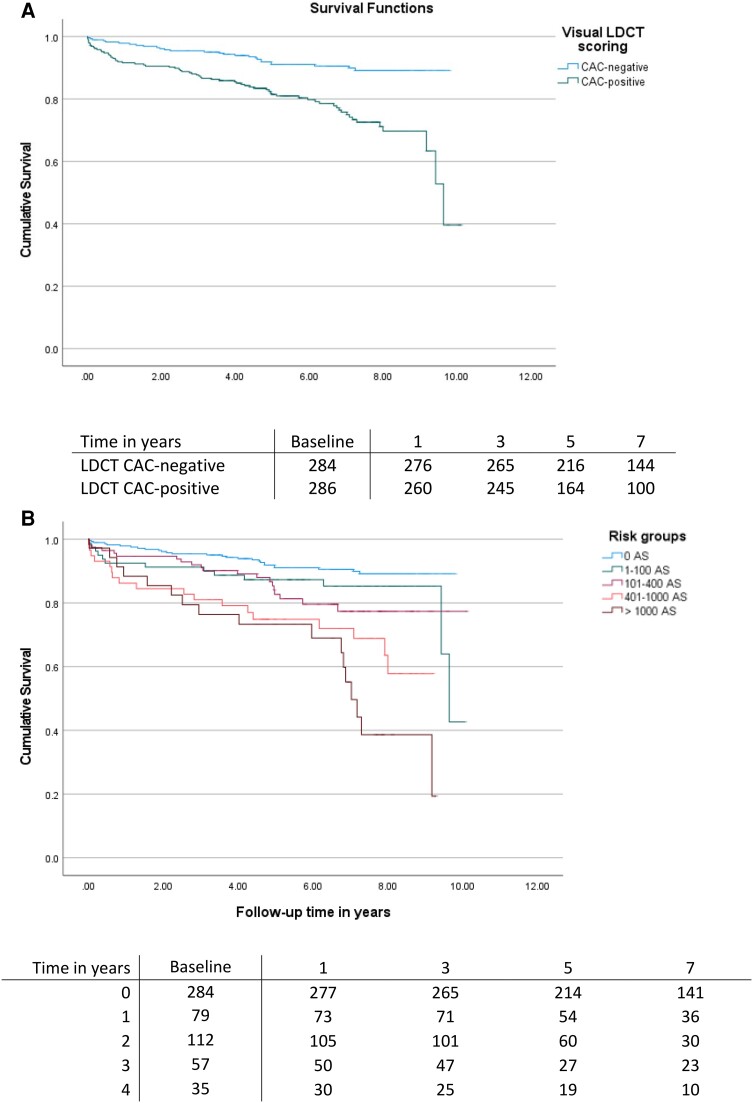
Major adverse cardiovascular event rate during follow-up in (*A*) in calcium-positive and calcium-negative LDCT groups and (*B*) risk groups based on visual calcium scoring from LDCT scans. Risk groups were defined as follows: 0, 0 AS; 1, 1–100 AS; 2, 101–400 AS; 3, 401–1000 AS; and 4, >1000 AS.^7^

**Figure 4:**
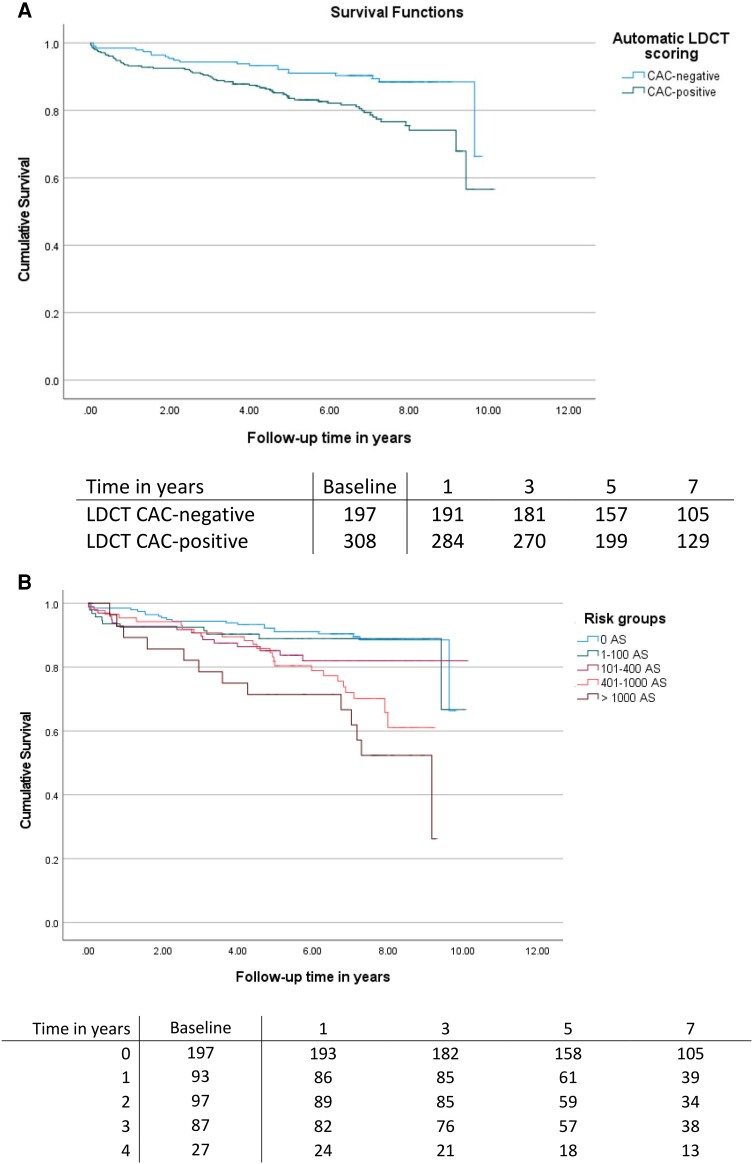
Major adverse cardiovascular event rate during follow-up in (*A*) calcium-positive and calcium-negative LDCT and (*B*) risk groups based on automatic calcium scoring from LDCT scans. Risk groups were defined as follows: 0, 0 AS; 1, 1–100 AS; 2, 101–400 AS; 3, 401–1000 AS; and 4, >1000 AS.^7^

The prognostic value of CAC scored visually on LDCT is depicted by Kaplan–Meier curves in *Figure 3*. Patients with visual CAC-positive LDCT were at higher risk of MACE as compared with patients with a CAC-negative LDCT (log-rank *P* < 0.001). This was especially marked in patients with visual CAC > 100 (log-rank *P* = 0.007) (*Figure 3B*). Based on automatic analysis, similarly to visual LDCT scoring, CAC-positive patients were characterized by higher MACE rates (*P* = 0.002; *Figure 4A*), especially for CAC > 400 (log-rank *P* < 0.001) (*Figure 4B*). The prognostic value of manual scoring on CACS is depicted in Supplementary data online, *Figure S2*.

### Combined LDCT CAC and PET

**Figure 5:**
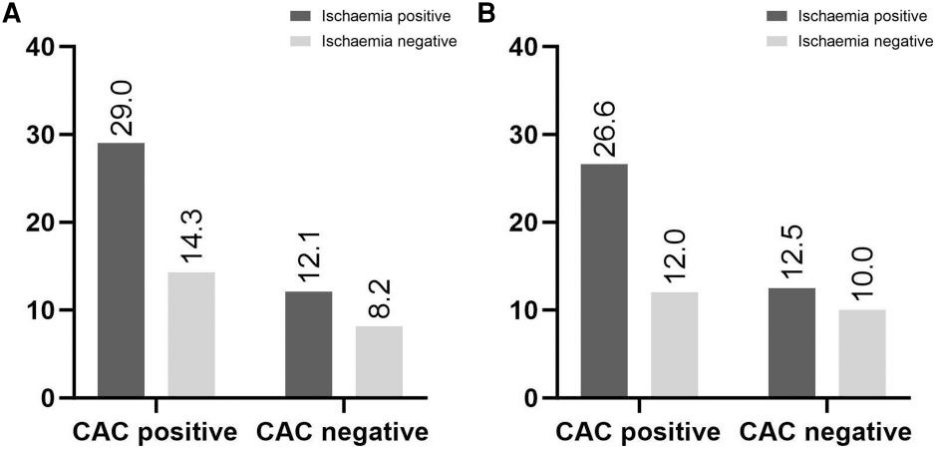
Percentage of MACE by ischaemia on ^15^O-water-PET scan and calcium on LDCT scans detected visually (*A*) and automatically (*B*), respectively.

**Figure 6:**
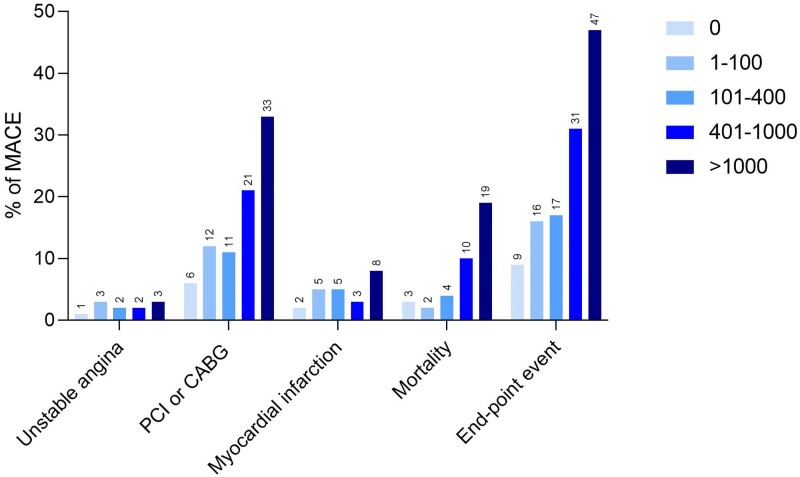
Summary of MACE by risk group based on visual calcium scoring from LDCT scans. Risk groups were defined as follows: 0, 0 AS; 1, 1–100 AS; 2, 101–400 AS; 3, 401–1000 AS; and 4, >1000 AS.^7^ PCI or CABG refers to late revascularizations, and endpoint event refers to all-cause death, myocardial infarction, late revascularization [percutaneous coronary intervention (PCI) and coronary artery bypass grafting (CABG)], or unstable angina.

The percentage of events was higher in patients with CAC-positive LDCT scan, irrespectively from the results of [^15^O]-water-PET (*Figure 5*). As depicted on *Figure 6*, the percentage of endpoint events increases with increasing CAC from LDCT, based on visual analysis. A univariable Cox models revealed increasing HR in each CAC risk categories, with significantly higher MACE in patients with CAC > 100, based on visual CAC scoring. When automatic scoring was applied, the significant increase in MACE was present in patients with CAC > 400 (*Table 5*). The univariable Cox model for the reference, manual CAC scoring from CSCT scans, is depicted in Supplementary data online, *Table S1*.

**Table 5:** Univariable Cox regression model to predict MACE for visual and automatic CAC scoring from LDCT scans

	Visual LDCT scoring			Automatic LDCT scoring		
	HR	95% CI	*P*	HR	95% CI	*P*
CAC risk groups						
0						
1–100	1.67	0.86–3.26	0.131	1.21	0.58–2.51	0.615
101–400	2.17	1.21–3.87	0.009	1.76	0.92–3.34	0.089
401–1000	3.96	2.18–7.19	<0.001	2.90	1.61–5.23	<0.001
>1000	6.24	3.39–11.47	<0.001	4.92	2.45–9.87	<0.001
^15^O-water-PET						
Ischaemia	2.45	1.62–3.84	<0.001	2.32	1.48–3.65	<0.001

Multivariable Cox models for MACE adjusted for perfusion defects from [^15^O]-water-PET scans and risk categorization based on visual and automatic CAC scoring from LDCT scans are summarized in *Table 6*. Both automatic and visual risk categorization from LDCT improved identification of patients at increased risk of MACE independently from myocardial perfusion results. For automatic CAC risk categorization from LDCT scans, patients with CAC > 400 were at increased risk of MACE. Based on visual CAC scoring from LDCT, CAC > 100 was associated with increased risk of MACE.

**Table 6:** Multivariable Cox regression models adjusted for perfusion defects defined on ^15^O-water-PET scans, to predict MACE for visual and automatic CAC scoring from LDCT scans

	Visual LDCT scoring			Automatic LDCT scoring		
	HR	95% CI	*P*	HR	95% CI	*P*
CAC risk group						
0						
1–100	1.51	0.77–2.98	0.224	1.12	0.54–2.34	0.760
101–400	1.91	1.06–3.45	0.032	1.58	0.82–3.04	0.174
401–1000	3.13	1.67–5.89	<0.001	2.30	1.23–4.29	0.009
>1000	4.77	2.49–9.15	<0.001	3.77	1.81–7.84	<0.001
^15^O-water-PET						
Ischaemia	1.66	1.04–2.65	0.034	1.70	1.05–2.76	0.032

Based on multivariable Cox regression analysis of associations of MACE with CAC calculated from CSCT and MPI defects, increased CAC was associated with an increased risk of MACE, which was significant for patients with CAC > 0 (see Supplementary data online, *Figure S2*). Harrel’s *C* statistics revealed that the CAC calculated from CSCT scans (Model 1) improved risk stratification more than that using CAC from LDCT (Model 2 and Model 3) (*C*-index, 0.74 vs. 0.70, *P* = 0.004, and 0.74 vs. 0.69, *P* = 0.004 for model 1: [^15^O]-water-PET and CSCT scan, model 2: [^15^O]-water-PET and visual scoring from LDCT scans, and model 3: [^15^O]-water-PET and automatic scoring from LDCT scans, respectively; Supplementary data online, *Figure S3*).

## Discussion

This study assessed the prognostic value of CAC scored visually and automatically on LDCT attenuation correction scans which were acquired during [^15^O]-water-PET imaging. It was found that visually and automatically determined CAC scoring from LDCT scans have an incremental prognostic value, irrespective from myocardial [^15^O]-water-PET results. If added to MPI-PET, CAC further improves the recognition of patients at high cardiovascular risk. Our findings strongly support reporting CAC from LDCT scans in addition to perfusion results from [^15^O]-water-PET.

Coronary calcium, which reflects the burden of atherosclerosis, plays a crucial role in cardiovascular risk stratification. Despite the indisputable importance of CAC in cardiovascular risk assessment, CSCT scans are not routinely performed before MPI-PET scans. However, LDCT scans routinely acquired during MPI might be utilized for CAC scoring and thereby making standard CSCT scans obsolete. As commonly known, CAC is calculated following the strictly defined Agatston method which should not be applied on scans other than CSCT scans.^5^ One of the attempts were made by Kaster *et al.*^18^ and Mylonas *et al.*,^19^ who changed the threshold of calcium definition from 130 to 50 HU. However, this approach not only increases noise and creates false positive results but also substantially hampers the idea of weighing calcium voxels based on the maximal HU number.^5^ Moreover, as Hounsfield values depend on tube voltage, threshold recalculation based on acquisition parameters is needed.^20^ Recently, an American expert consensus proposed a visual scoring of non–ECG-gated and non-contrast scans as a method of choice.^21^ This simple, cost-effective, and elegant way of calcium assessment from LDCT was introduced by Einstein *et al.*,^7^ who analysed LDCT acquired with SPECT-CT and reported 63% of agreement within the same risk category, while Engbers *et al.*^22^ reported an agreement of 71% with CSCT scans. In our study, the agreement within the same risk category was lower (56.6%), also as compared with our previous analysis (74%)^23^ which might be explained by a greater amount of motion artefacts on LDCT scans analysed in the present study, lack of the breath-hold during the scan, and greater slice thickness. Nevertheless, 84.1% of analysed scans did not vary by more than one category and majority of scans was correctly defined as CAC-positive, leading to a specificity of 97.6%. Therefore, visual CAC scoring from LDCT scans correctly defined patients with greater amount of coronary calcium.

As specified in guidelines, the optimal method of CAC scoring from LDCT scans still has to be defined.^6^ This, in addition to great improvement in AI methods, led to a proliferation of studies focused on the development of automatic CAC scoring method from non–ECG-triggered, non-contrast scans. Lessmann *et al.*^24^ presented a method for automatic cardiovascular risk categorization based on LDCT scans acquired for lung cancer screening, which enabled reliable cardiovascular risk assessment. Van Velzen *et al.*^8^ applied the same deep learning algorithm^24^ for calcium scoring from different types of chest CT scans, including LDCT acquired in PET/CT studies, and gained a good agreement with the manual calcium scoring from these LDCT scans (κ 0.92, 95% CI: 0.91–0.93). Another interesting approach was presented by Zeleznik *et al.*,^9^ who created a deep learning system predicting cardiovascular events based on the presence and amount of coronary artery calcium in asymptomatic individuals. Importantly, in none of these studies, an automatic scoring from LDCT results was compared with a reference, CSCT scans. Recently, Pieszko *et al.*^10^ presented a deep learning algorithm which calculates CAC from LDCT acquired with PET scans and compared these results to dedicated CSCT scans gaining a good agreement (linearly weighted κ 0.62, 95% CI: 0.6–0.64), which is comparable with the results of visual and automatic scoring presented in our study [κ 0.66 (95% CI: 0.62–0.70); κ 0.58 (95% CI: 0.53–0.62), respectively]. The discrepancy in the agreement between studies applying only LDCT scans^8,9,24^ and those which validated LDCT on CSCT scans should be attributed to the presence of a reference CT^10^ scan in the latter ones.

In our study, both the visual and automatic method resulted in an underestimation of Agatston scores and occasional misclassification of patients as CAC-negative, which is in line with earlier observations.^7,9,10,22^ This yielded a lower sensitivity and NPV and brought the greatest limitation of CAC scoring from LDCT scans to light. Based on European guidelines, patients with CAC > 100 are considered as individuals of higher cardiovascular risk.^25^ Importantly, based on our analysis, CAC scoring from LDCT sufficiently defines this group of patients. As presented by Blaha *et al.*,^26^ the absence of CAC is associated with lower incidence of cardiovascular events. Therefore, the greatest limitation of CAC scoring from LDCT scans remains its inability to detect small, low-density calcifications. In our analysis, based on automatic scoring, 14.4% patients were incorrectly defined as CAC-negative, while based on visual scoring, 21.8% of patients was misclassified as CAC-negative, which is comparable with results reported by the group of Einstein (22%).^7^ Low sensitivity of LDCT in CAC detectability might be considered as the main weak point of LDCT scoring. Nevertheless, as shown in our study, despite this limitation, CAC scoring from LDCT still enables to determine patients with non-obstructive atherosclerotic disease which is not feasible with MPI-PET alone.

This study was designed with the aim to assess the clinical value of CAC from LDCT acquired during MPI [^15^O]-water-PET scans in relation with MACE. The findings observed in this study mirror those of previous studies that have examined the clinical value of CAC assessed from LDCT in addition to MPI.^10,27–29^ Gaibazzi and colleagues^27^ demonstrated that visually detected calcium on LDCT acquired during MPI-SPECT was an independent predictor of MI and all-caused death. Trpkov and colleagues^28^ applied simple visual scoring, in which CAC was defined as absent, present, or extensive, and also found that this was a predictor of MACE independent from the results of MPI-SPECT. Dekker and colleagues^29^ validated an automatic calcium scoring method from LDCT scans acquired during ^82^Rb-PET, which resembled high calcium (>400 AS) from low calcium (<400 AS) patients, and high calcium was also independently from [^82^Rb]-PET results associated with MACE. The current study further confirms the association between CAC and MACE in symptomatic patients. The detection of CAC on LDCT scan, either visually or automatically, helps to define patients at increased risk of MACE, even in the absence of ischaemia on PET. Additional risk stratification further improved the prediction of MACE independently from myocardial perfusion results derived from gold standard [^15^O]-water-PET scans.

Undoubtedly, our analysis underscores the superior performance of CAC from CSCT over CAC from LDCT in terms of improved risk prediction. Nevertheless, in situations where CSCT scans are unavailable, it is advantageous to incorporate CAC analysis from LDCT scan in clinical decision-making, as it demonstrated enhanced ability to predict event risks compared with myocardial perfusion scan alone.

The fact that CAC is an independent predictor of MACE is an important finding strongly supporting to report CAC findings from LDCT scans, which in addition to a very low radiation dose of both scans creates unique information about cardiovascular risk. This is a strong message to patients with no ischaemia on MPI, who might be still at increased risk of cardiovascular events.

### Study limitations

Our study has several limitations. First, due to the relatively small sample, results of automatic CAC scoring should be further investigated. On the other hand, [^15^O]-water-PET scans are not widely used in everyday clinical practice, which makes this patient cohort unique. Second, this was a single-centre study, and all LDCT scans were acquired with the same protocol on the same scanner. Therefore, automatic CAC scoring method should be further validated on scans acquired with different vendors. Third, in this study, a 5 instead of 3 mm slice thickness of LDCT scans was applied. However, as presented results demonstrated a good agreement with a reference CSCT scan, we expect that for 3 mm slice thickness, the visual and automatic scoring will further improve in terms of CAC detection and risk group categorization. Fourth, the correspondence between the localization of calcifications detected on LDCT scans and CSCT scans was not investigated. Therefore, only patient-based analysis and not lesion-based analysis was performed.

## Conclusion

The agreement in risk classification between visual as well as automatic CAC scoring from LDCT and reference CSCT enables improved MACE risk classification. Patients with a CAC-positive LDCT based on visual or automatic LDCT scoring have higher MACE rates. Visual CAC scoring from LDCT has better specificity to detect CAC as compared with automatic scoring. Visual and automatic CAC scoring from LDCT are able to more accurately identify patients at risk for long-term MACE, independently from [^15^O]-water-PET MPI. Therefore, we advise routine CAC scoring from LDCT scans in addition to MPI-PET in symptomatic patients, when CSCT scan is not available.

## Supplementary data

Supplementary data are available at *European Heart Journal - Cardiovascular Imaging* online.

## Supplementary Material

jeae081_Supplementary_Data

## Data Availability

The data underlying this article will be shared on reasonable request to the corresponding author.
