# Supplementary material for: The prognostic value of visual and automatic coronary calcium scoring from low-dose computed tomography-[15O]-water positron emission tomography
Source: Eur Heart J Cardiovasc Imaging. 2024 Mar 26;25(9):1186–96. doi: 10.1093/ehjci/jeae081 (PMC11346363; doi:10.1093/ehjci/jeae081)
Supplement: jeae081_Supplementary_Data [file jeae081_supplementary_data.docx]

# Supplementary material

# Methods

## Study design

Of 742 patients, 57 were excluded due to uninterpretable LDCT images, 15 due to inconclusive PET results, and 72 were lost to follow-up. In addition, 26 patients, who had a PCI or CABG in the past, were excluded due to LDCT artifacts. In total, 572 patients were included in the analysis. None of the included patients had an event or underwent a revascularization between the [^15^O]-water-PET and the CSCT scan. The study complied with the Declaration of Helsinki.

### Image acquisition^15^O-water- PET and LDCT

Images were acquired on a Gemini TF 64 PET/CT-scanner (Philips Healthcare, Best, The Netherlands). A dynamic perfusion scan was performed during rest and adenosine stress (140 ug/kg/min) induced hyperaemia using 370 MBq [^15^O]-water as radioactive tracer. Parametric images of myocardial blood flow (MBF) were generated using in-house developed software (CardiacVUer, Amsterdam UMC: Vrije Universiteit Amsterdam)^1^. Vascular territories were defined according to the standardized 17-segment model of the American Heart Association^2^. A hyperaemic MBF ≤ 2.3 ml/min/g in 2 adjacent segments (out of 17) within one vascular territory was considered indicative of myocardial ischemia^3^. Patients were instructed to abstain from products containing caffeine 24 hours prior to the PET scan. Hyperaemic MBF was used for analysis because of its greater ability to identify ischaemia and better prognostic value compared to CFR or resting MBF using ^15^O-water as tracer^3^.

LDCT scans were acquired on a Gemini TF 64 PET/CT-scanner (Philips Healthcare, Best, The Netherlands) and performed after rest and adenosine induced stress, resulting in two LDCT scans per patient. LDCT scans were non-ECG-triggered, non-contrast, free breathing without breath-hold. All patients were scanned at 120 kVp. Images were reconstructed with filtered back projection with a kernel B at 5 mm slice thickness and 5 mm increment and in-plane resolution 1.17mm. The dose of LDCT ranged from 0.2-0.3 mSv

### CSCT – reference calcium scan

CSCT scans were prospectively ECG-triggered in diastolic phase without IV contrast enhancement, during inspiratory breath-hold, acquired on PET/CT system (Hybrid PETCT 64 Gemini) and CT system (Philips Brilliance iCT, Philips Healthcare, Best, the Netherlands) with a tube voltage of 120 kVp. The dataset was reconstructed using a kernel B at 2.5 mm slice thickness with an increment of 2.5 mm and in-plane resolution 0.37 mm.

## Visual scoring from LDCT scans

Visual scoring was performed by one observer (M.M.D.), who was blinded to the CSCT scoring results. LDCT scans were analysed on a per patient level and considered as CAC-positive if at least one of the LDCT scans (rest or stress) was CAC-positive. Conversely, to consider the LDCT scan as CAC-negative, both the rest and stress LDCT scans had to be CAC-negative. If the visual CAC score between rest and stress LDCT scans differed, the higher risk group category was used. The example of risk groups based on visual scoring is presented on supplementary figure 1.

## Automatic scores

Briefly, to enable calcium quantification in LDCT scans with large slice thickness, low image resolution and cardiac motion artifacts, the automatic scoring does not apply the standardly used 130 HU threshold for calcium detection. Instead, it mimics visual scoring to define the presence and amount of CAC on LDCT scans. Using a generative adversarial approach, the method decomposes an image slice containing CAC into its counterpart without CAC and a CAC-map, which reflects the amount and location of CAC (Figure 2). As LDCT scans are not calibrated for quantification of CAC mass, we calculated CAC pseudomass. The CAC pseudomass is calculated by summing the HU values of CAC voxels and multiplying by the voxel spacing and it is uncalibrated CAC mass. 136 LDCT scans from 68 patients with suspected CAD from this cohort were previously included in the model training set and were therefore excluded from analyses. The algorithm was used to obtain CAC-maps for the LDCT evaluation set of 507 patients, which were then used to quantify CAC into CAC mass.

If the automatic score between the rest and stress LDCT scan differed, the higher risk group category was included for analysis. Additionally, scans were categorized as CAC-positive or CAC-negative based on the automatic score.

### CAC scoring from reference CSCT scans

Manual CAC scoring of CSCT scans, defined as the reference, was performed according to the Agatston method, in which calcium was defined using a standard threshold of 130 HU and area ≥ 1mm^2^ and calculated using a designated software (IntelliSpace, Philips Health Care, Best, The Netherlands)^4^.

## Study endpoint

Events were defined according to the current clinical guideline^5^. The first and most serious event was considered as the event of the patient, with death being considered more serious than non-fatal myocardial infarction.

# Tables and figures

Supplementary Figure 1 Univariable and Multivariable Cox regression models adjusted for perfusion defects defined on ^15^O-water – PET scans, to predict MACE for visual and automatic CAC scoring from the reference, CSCT scans.

|  | Reference - manual CAC scoring from CSCT scans | | | | | |
| --- | --- | --- | --- | --- | --- | --- |
|  | Univariable Cox regression | | | Multivariable Cox regression | | |
|  | HR | 95% CI | P | HR | 95% CI | P |
| CAC risk groups |  |  |  |  |  |  |
| 0 | - | - | - | - | - | - |
| 1-100 | 4.30 | 1.87 - 9.91 | < 0.001 | 4.00 | 1.72 - 9.28 | < 0.001 |
| 101-400 | 5.27 | 2.29 - 12.58 | < 0.001 | 4.72 | 1.98 - 11.31 | < 0.001 |
| 401-1000 | 9.87 | 4.07 - 23.91 | < 0.001 | 8.43 | 3.38 - 20.98 | < 0.001 |
| >1000 | 13.87 | 5.92 - 32.49 | < 0.001 | 11.17 | 4.55 - 27.43 | < 0.001 |
| ^15^O-water – PET |  |  |  |  |  |  |
| Ischemia | 2.45 | 1.62 – 3.84 | < 0.001 | 1.40 | 0.88 – 2.23 | 0.152 |
|  |  |  |  |  |  |  |


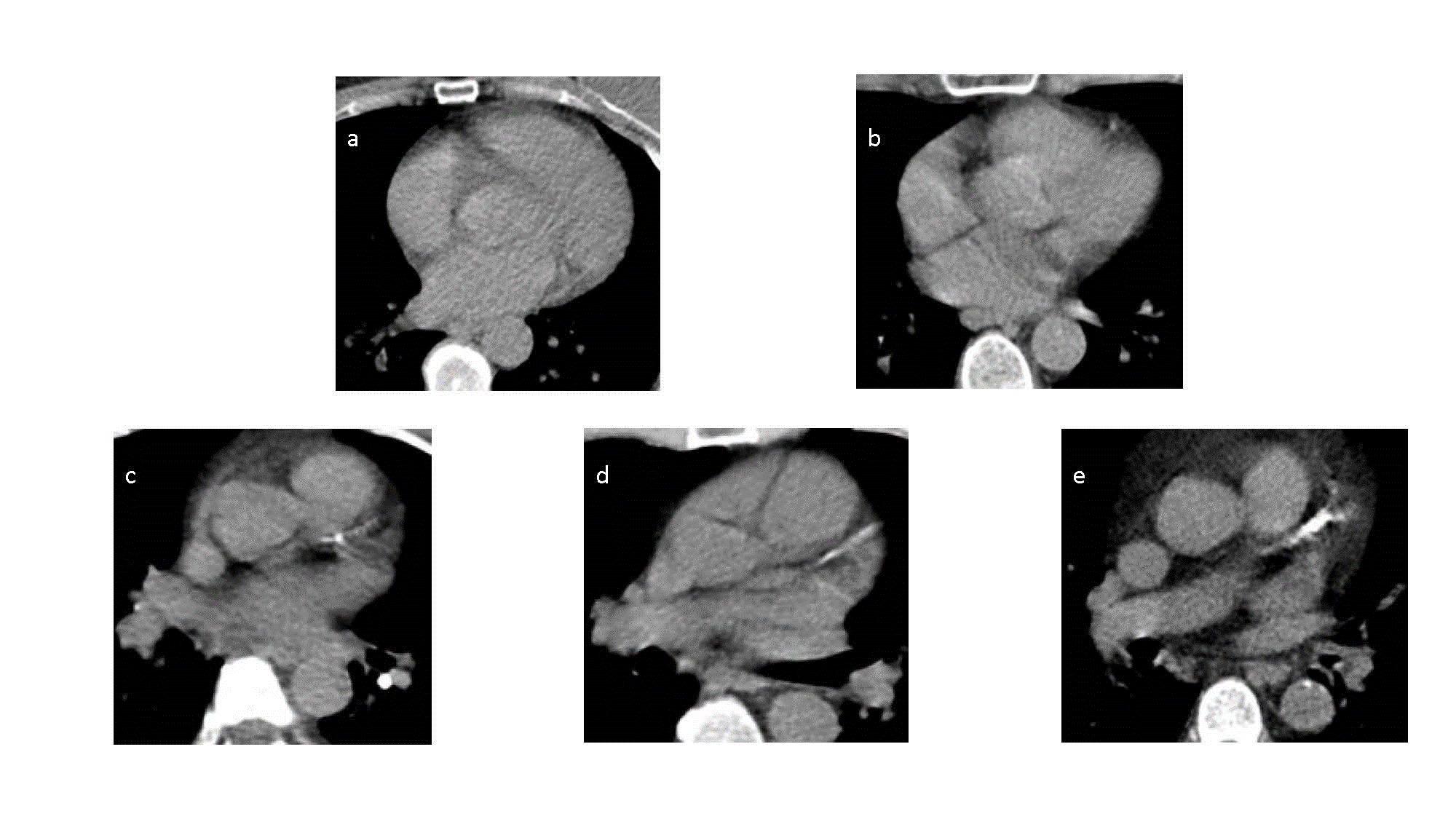


Supplementary figure 1 Example of visual assessment of coronary artery calcium from low dose CT scans. Agatston scores risk groups were defined as follows: 0: 0 AS; 1: 1-100 AS; 2: 101-400 AS; 3: 401-1000 AS; 4: >1000 AS^6^


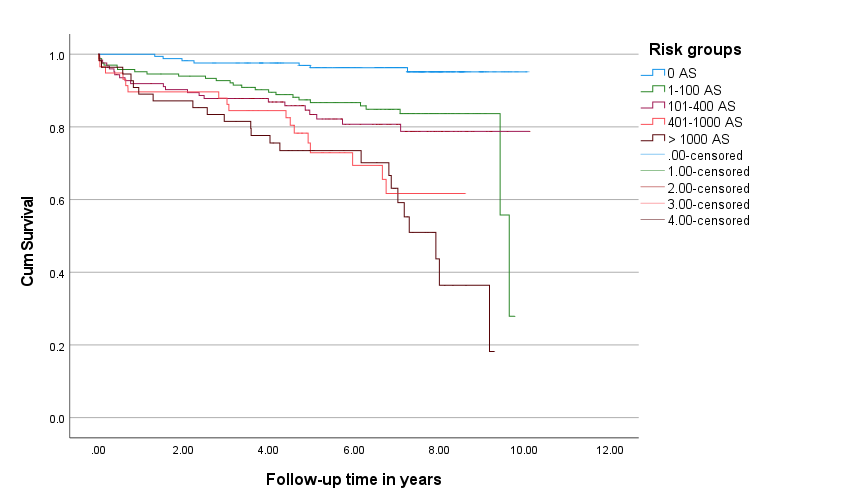

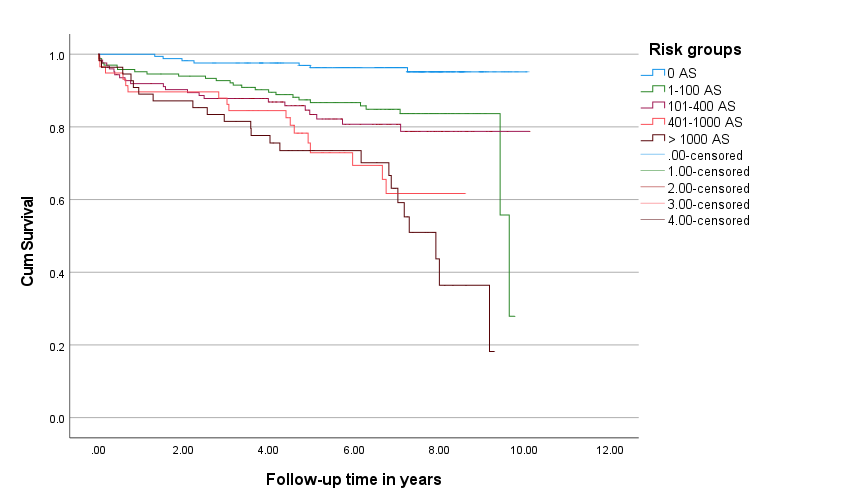


| Time in years | Baseline | 1 | 3 | 5 | 7 |
| --- | --- | --- | --- | --- | --- |
| 0 | 166 | 166 | 160 | 141 | 93 |
| 1 | 166 | 156 | 150 | 114 | 73 |
| 2 | 123 | 111 | 105 | 67 | 43 |
| 3 | 57 | 52 | 49 | 26 | 14 |
| 4 | 55 | 46 | 42 | 27 | 15 |

Supplementary Figure 2 Major adverse cardiovascular events rate during follow-up in risk groups based on reference, manual calcium scoring from CSCT scans.

Agatston scores risk groups were defined as follows: 0: 0 AS; 1: 1-100 AS; 2: 101-400 AS; 3: 401-1000 AS; 4: >1000 AS^6^.


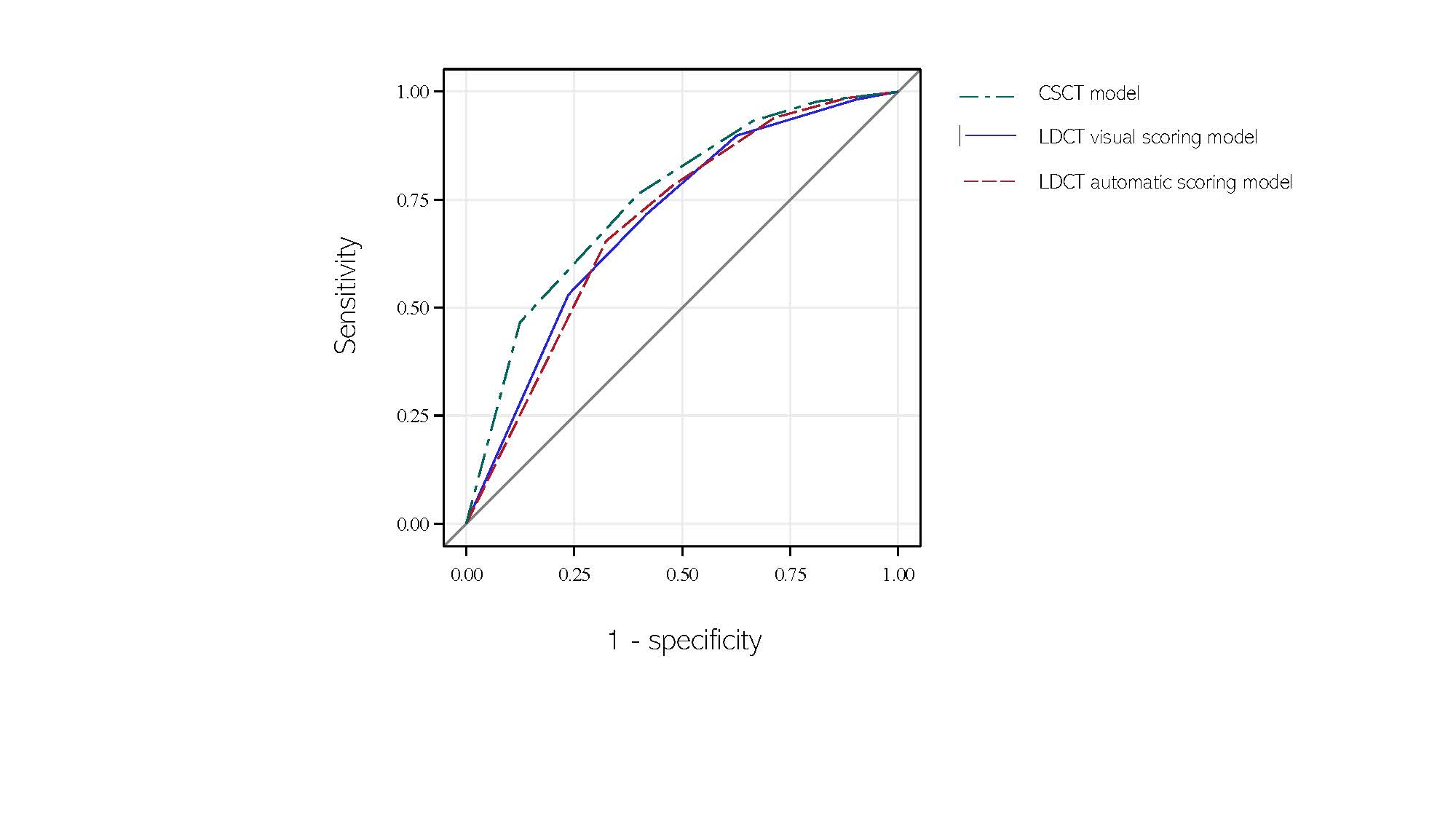


Supplementary figure 3 The depiction of ROC curves comparing risk prediction of three multivariable Cox regression models: model 1: [^15^O]-water-PET and CSCT scan (AUC 0.74, 95%CI 0.71-0.79), model 2: [^15^O]-water-PET and visual scoring from LDCT scans (AUC 0.70, 95% CI: 0.65-0.75), and model 3: [^15^O]-water-PET and automatic scoring from LDCT scans (AUC 0.69, 95%CI 0.65 – 0.74), respectively

# References

1. Harms HJ, Knaapen P, Haan S De, Halbmeijer R, Lammertsma AA, Lubberink M. Automatic generation of absolute myocardial blood flow images using [ 15O]H2O and a clinical PET/CT scanner. *Eur J Nucl Med Mol Imaging* 2011;**38**:930–939.

2. Cerqueira MD, Weissman NJ, Dilsizian V, Jacobs AK, Kaul S, Laskey WK, Pennell DJ, Rumberger JA, Ryan TJ, Verani MS. Standardized myocardial segmentation and nomenclature for tomographic imaging of the heart. *J Cardiovasc Magn Reson* 2002;**4**:203–210.

3. Bom MJ, Diemen PA van, Driessen RS, Everaars H, Schumacher SP, Wijmenga J-T, Raijmakers PG, Ven PM van de, Lammertsma AA, Rossum AC van, Knuuti J, Danad I, Knaapen P. Prognostic value of [15O]H2O positron emission tomography-derived global and regional myocardial perfusion. *Eur Hear journal Cardiovasc Imaging* 2020;**21**:777–786.

4. Agatston AS, Janowitz WR, Hildner FJ, Zusmer NR, Viamonte M, Detrano R. Quantification of coronary artery calcium using ultrafast computed tomography. *J Am Coll Cardiol* 1990;**15**:827–832.

5. Knuuti J, Wijns W, Achenbach S, Agewall S, Barbato E, Bax JJ, Capodanno D, Cuisset T, Deaton C, Dickstein K, Edvardsen T, Escaned J, Funck-Brentano C, Gersh BJ, Gilard M, Hasdai D, Hatala R, Mahfoud F, Masip J, Muneretto C, Prescott E, Saraste A, Storey RF, Svitil P, Valgimigli M, Windecker S, Aboyans V, Baigent C, Collet JP, Dean V, Delgado V, Fitzsimons D, Gale CP, Grobbee DE, Halvorsen S, Hindricks G, Iung B, Jüni P, Katus HA, Landmesser U, Leclercq C, Lettino M, Lewis BS, Merkely B, Mueller C, Petersen S, Petronio AS, Richter DJ, Roffi M, Shlyakhto E, Simpson IA, Sousa-Uva M, Touyz RM, Benkhedda S, Metzler B, Sujayeva V, Cosyns B, Kusljugic Z, Velchev V, Panayi G, Kala P, Haahr-Pedersen SA, Kabil H, Ainla T, Kaukonen T, Cayla G, Pagava Z, Woehrle J, Kanakakis J, Toth K, Gudnason T, Peace A, Aronson D, Riccio C, Elezi S, Mirrakhimov E, Hansone S, Sarkis A, Babarskiene R, Beissel J, Cassar Maempel AJ, Revenco V, Grooth GJ de, Pejkov H, Juliebø V, Lipiec P, Santos J, Chioncel O, Duplyakov D, Bertelli L, Dikic AD, Studencan M, Bunc M, Alfonso F, Back M, Zellweger M, Addad F, Yildirir A, Sirenko Y, Clapp B. 2019 ESC guidelines for the diagnosis and management of chronic coronary syndromes. *European Heart Journal*.

6. Einstein AJ, Johnson LL, Bokhari S, Son J, Thompson RC, Bateman TM, Hayes SW, Berman DS. Agreement of visual estimation of coronary artery calcium from low-dose CT attenuation correction scans in hybrid PET/CT and SPECT/CT with standard Agatston score. *J Am Coll Cardiol* 2010;**56**:1914–1921.
